# A credit risk assessment model of borrowers in P2P lending based on BP neural network

**DOI:** 10.1371/journal.pone.0255216

**Published:** 2021-08-03

**Authors:** Zhengwei Ma, Wenjia Hou, Dan Zhang

**Affiliations:** School of Economics and Management, China University of Petroleum- Beijing, Beijing, China; University of Brescia, ITALY

## Abstract

Peer-to-Peer (P2P) lending provides convenient and efficient financing channels for small and medium-sized enterprises and individuals, and therefore it has developed rapidly since entering the market. However, due to the imperfection of the credit system and the influence of cyberspace restrictions, P2P network lending faces frequent borrower credit risk crises during the transaction process, with a high proportion of borrowers default. This paper first analyzes the basic development of China’s P2P online lending and the credit risks of borrowers in the industry. Then according to the characteristics of P2P network lending and previous studies, a credit risk assessment indicators system for borrowers in P2P lending is formulated with 29 indicators. Finally, on the basis of the credit risk assessment indicators system constructed in this paper, BP neural network is built based on the BP algorithm, which is trained by the LM algorithm (Levenberg-Marquardt), Scaled Conjugate Gradient, and Bayesian Regularization respectively, to complete the credit risk assessment model. By comparing the results of three mentioned training methodologies, the BP neural network trained by the LM algorithm is finally adopted to construct the credit risk assessment model of borrowers in P2P lending, in which the input layer node is 9, the hidden layer node is 11 and output layer node is 1. The model can provide practical guidance for China and other countries’ P2P lending platforms, and therefore to establish and improve an accurate and effective borrower credit risk management system.

## 1. Introduction

2013 is known as the first year of China’s Internet finance. With the comprehensive development of big data, blockchain Internet technologies such as mobile payment, the traditional service business and operation mode of financial system are constantly changing. At present, the Chinese Internet financial business mainly includes the third-party payment, crowdfunding, P2P lending, digital currency, big data finance and others, among which the P2P lending has developed rapidly. With trading volume reaching 964.911 billion yuan in 2019, the online lending sector ranked first in the world, the growth rate of which was 293% compared to 329.194 billion yuan in the early 2014. (source: P2Peye.com). However, as an emerging mode of loan financing, P2P lending has become increasingly problematic in terms of risk exposure due to its lack of complete mechanism of risk control and early warning. In fact, China’s online lending has suffered from serious borrower credit risk problems, and many platforms fail to earn profits due to high bad debt rates. According to the statistics of National Internet Finance Association of China (NIFA), the average overdue rate in China’s P2P lending platforms was 3.27% in 2019. By establishing scientific credit risk assessment indicators system and model, this paper provides effective ways to solve the problem of borrower credit risk.

In this paper, authors focus on the credit risk assessment of borrowers in P2P lending. By summarizing and analyzing the existing research, combining the characteristics of P2P lending industry, authors establish a set of P2P lending borrower credit risk assessment indicators system, which is applicable to China’s online lending. Also, authors construct a credit risk assessment model of borrowers in P2P lending based on BP neural network.

## 2. Literature review

So far, researches on credit risk assessment of individuals in P2P lending from home and abroad mainly focus on two aspects, credit risk assessment indicators and assessment methods.

In terms of online lending credit risk assessment indicators, Zhang, etc. (2012) [22] conducted a research on FICO, American individual credit scoring system, and concluded that FICO evaluates users’ credit by examining credit repayment history, the number of credit accounts, credit service life, types of credit in use, and assessment of new credit accounts opened, with ratios of 35%, 30%, 15%, 10%, and 10%, respectively [1]. Tan, etc. (2017) studied the influence of loan requests and borrower defaults on the credit risk of P2P lending borrowers by analyzing 8 indicators: total loan requests, credit limit, loan request status, unpaid loan principal and interests, repayment term, annual interest rate, credit rating, and total loan amount [2]. Emektera, etc. (2015) noted that indicators such as credit rating, debt-to-income ratio, and FICO credit score can all be applied to assess the default risk of P2P lending borrowers. It shows that a borrower with lower credit rating, higher debt-to-income ratio, and lower FICO credit score has a higher risk to default [3]. Xiao, etc. (2015) analyzed the correlations among following indicators: age and gender, credit rating, number of successful and unsuccessful borrowings, repayment interest rate, repayment term and borrower credit risk. And it is found that all indicators except credit rating can effectively predict borrower credit risk control, while credit rating sometimes even has a negative impact on prediction [4]. Wang and Liao (2014) believe that the behavior of P2P lending borrower is affected by borrower authentication indicators and mode [5].

To analyze borrower credit risk in a more comprehensive way, some scholars have incorporated ‘soft information’, such as information in real life and online social network of borrower, into the assessment of borrower credit risk. Yang, etc. (2018) believe that by introducing borrower’s social network information to identify credit risk and to form default constraints is conducive to reducing information asymmetry in P2P lending [6]. Carlos, etc. (2015) found that the risk level prediction provided by the Lending Club platform has reference value based on the logistic regression model analysis. But its prediction only includes the most predictive default factors. Therefore, the establishment of a risk prediction model that comprises more indicators is helpful to improve the accuracy of prediction and to avoid investors’ misjudgment [7]. Zhang, etc. (2016) constructed a credit scoring model for P2P lending that incorporates social media information. The analysis found that lending information, social media information, and credit information are important factors in predicting defaults. On the contrary, platform credit ratings did not work effectively in predicting [8]. Ma, etc. (2018) assessed the credit risk of borrowers through their phone usage data and studied the relationship between phone usage patterns and lending default behavior [9].

As one of ‘soft information’ that is easily accessible and informative, the loan description has attracted more and more attention from scholars. Iyer, etc. (2009) believe that ‘soft information’ can help identify lenders’ credit scores [10]. Xin, etc. (2017) studied the relationship between the borrower’s personality tendencies embodied in loan descriptions and credit risk through extracting information about the personality tendency of P2P lending borrowers [11]. Chen, etc. (2018) studied the role of punctuation in P2P lending market. Their study found that the number of punctuation marks in a borrower’s loan description exert an influence on credit risk. The credit reputation of borrower can be identified by the application of punctuation in loan descriptions [12]. Jiang, etc. (2017) proposed a prediction method that combines traditional credit assessment features with ‘soft features’ extracted from descriptive loan information. The model selects relevant features from descriptive loan information based on the LDA (Latent Dirichlet Allocation) model and combines them with traditional assessment features to predict lender default by means of a two-dimensional feature selection [13].

Currently, establishing credit risk assessment models is mainly based on statistical and econometric methods, as well as machine learning. Statistical and econometric methods mainly quantifies borrower’s credit risk assessment by constructing models to describe the functional relation of risk warning issue. Due to the fact that machine learning does not require priori assumptions, pattern recognition methods such as decision tree classifiers and neural network classifiers are increasingly applied to establishing credit risk assessment models. Li, etc. (2016) studied abnormal creditors through the method of outlier test. The result showed that when K = 5, the outlier had a low credit score and the model achieved the highest accuracy in prediction [14]. Zhang, etc. (2017) developed a credit risk assessment model based on flexible neural tree (FNT). Through comparison it is found that the credit risk assessment model based on FNT performed better [15]. Li, etc. (2013) believe that there are shortcomings in traditional credit assessment methods. Therefore, they developed a personal credit assessment model based on sparse Bayesian learning. By comparison, it is found that the classification accuracy of this model is better than traditional methods such as K-nearest neighbor algorithm and Naive Bayes [16]. Guo, etc (2016) added a certain weight to a borrower’s past loans through kernel logistic regression. Then the credit risk is quantitatively rated by the time difference between the past loans and the latest loans to predict the borrower’s credit standing [17]. Malekipirbazari and Aksakalli (2015) established an assessment model for predicting borrowers’ credit risk through random forests(RF) algorithm. Its prediction result is found better than FICO scores and LC platform ratings [18]. Ye, etc. (2018) proposed Random Forests Optimized by Genetic Algorithms and Profit Scores (RFoGAPS) to further improve the prediction accuracy of random forests [19]. Ding and Luo (2017) found that the application of Stacking integration strategy can significantly reduce the proportion of Type I and Type II errors compared with single machine learning method, with its prediction accuracy higher than single model [20]. Jiang, etc.(2014) optimized the weights of characteristic variables in the case base by using BP neural network, logistic regression. Therefore, the accuracy and interpretability of the model were improved [21]. Zhu C and Zhu N (2017) utilized the five scale method to construct comparative matrix and determine the weights of credit risk indicators for P2P lending borrowers. The study further established a fuzzy evaluation model for credit risk based on fuzzy mathematics theory [22].

To improve the prediction accuracy of a model, some scholars applied the combination of two or more statistical methods to enhance credit risk model based on single method for online lending borrowers. Cao, etc. (2018) constructed 20 different ensemble-learning models by using data from Renrendai—one of the oldest online lending information intermediary service platforms in China, 4 ensemble-learning methods including Bagging, Boosting, etc. and 5 base classifiers such as LR and CART. By comparing the accuracy of model assessment, it is found that predictive ability of early warning system based on the Rotation Forest integrated model performed best [23]. Bai, etc. (2017) evaluated the personal credit in Internet micro-finance on the basis of random forest (RF) with Bagging-type algorithm, XGBoost with Boosting algorithm and Support Vector Machine (SVM) respectively. Then the study conducted simple weighted voting on the above three models via Blending integrated strategy, with a weighting ratio of 1: 8:1[24]. Li, etc. (2018) constructed a multi-round ensemble learning model based on heterogeneous ensemble framework to improve traditional credit risk assessment models. The ensemble learning model used different algorithms including XGBoost, Deep Neural Networks, and logistic regression model to significantly improve prediction accuracy compared to traditional machine learning and ensemble-learning models [25]. Xia, etc. (2017) put forward an ensemble-learning model that considers costs based on the characteristics of P2P lending. The model uses XGBoost algorithm to identify borrowers’ potential default risk [26]. Wang, etc. (2018) believe that borrower’s repayment behavior is dynamically evolving. Therefore, they proposed a novel scoring model based on EM-random forest (EMRF) algorithm. The model is used to predict the dynamic probability of borrower default over time in P2P lending. It is found to have outperformed standard hybrid model and logistic regression model in predicting monthly dynamic default probability [27].

## 3. Risk assessment model of BP neural network

### 3.1 Overview of BP neural network

**Fig 1:**
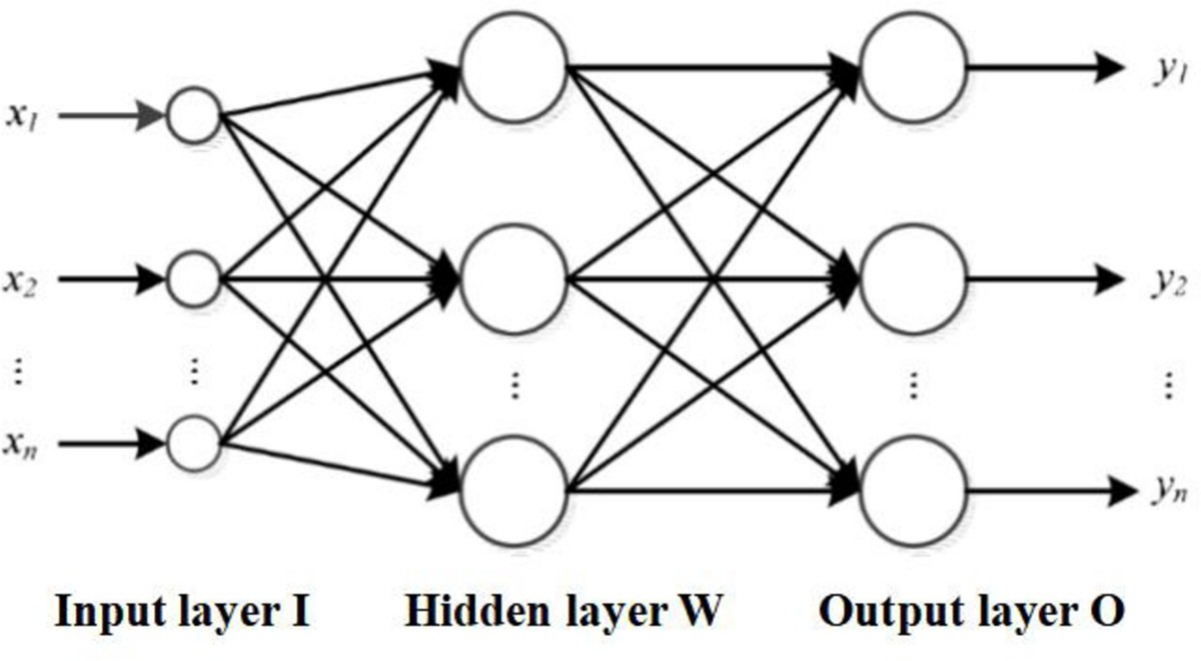
BP neural network model structure.

BP neural network is a neural network that uses the Error Back Propagation (BP) algorithm for learning, which was proposed by Rumelhart, McClelland, etc. The development of BP neural network solves the learning problems of multi-layer neural networks. BP neural network is a multilayer neural network with three or more layers, including several hidden layers in addition to input layer and output layer. The topological structure of a three-layer BP neural network is shown in **Fig 1**.

The BP neural network is a multi-layer structure, which helps the neural network to mine more information contained in the input samples and deal with more complex data processing. It uses an error back propagation algorithm for learning. The data enters the neural network from the input layer and propagates backwards through the hidden layer to the output layer. As the neural network trains the weights of the neurons, the transmitted signal propagates backwards in the direction of reducing errors, and the connection weights between the neurons of each layer are modified layer by layer from the output layer through the hidden layer. The output value modified by the error back propagation is again connected to the input neuron as the input for next calculation. In such an iteration cycle, the output value of the neural network is gradually reduced until it becomes stable.

### 3.2 Model construction based on BP neural network

Based on BP neural network, the credit risk assessment system for borrowers in P2P lending in this paper includes following content: Firstly, authors collect 2 dimension text features such as project title and loan descriptions about P2P lending borrowers and encode the text information with numbers, which then is transformed into non-textual information. Secondly, 28 dimension non-text features such as gender, age, education, and marital status are collected from the borrower’s registration information. Thirdly, in order to take both text and non-text features as input, authors combined the transformed textual with non-textual information. The neural network is then trained by cross-entropy loss function to multi-label the information of P2P lending borrower and design mapping relationship. Finally, based on the mapping relationship, authors obtain prediction result through output layer of the Sigmoid function, and predict the credit risk of borrower by binary classification. There are two types of results: being able to repay on time and not being able to repay on time. Therefore authors can assess the credit risk of borrower in P2P lending.

#### 3.2.1 The design of text feature module

Due to the fact that some of the projects in this experiment do not include information about lending purpose. Also, some borrowers choose incompatible purpose with the loan description. Therefore, to ensure the accuracy and effectiveness of the data, authors design text feature module to supplement and perfect the information related to borrower’s lending purpose. Borrower often mentions lending purpose in the loan description and loan title. The Ansj tokenizer can atomically segment the long text in the borrower’s loan description and loan title, and extract the borrower’s information such as lending purpose, city, etc., which is used as supplement to the missing information and adjustment to the wrong information. Ansj is a Chinese words tokenizer based on n-Gram+CRF+HFF. The concrete steps for Ansj to segment text features are as follows:

Step 1: Rough segmentation of text information based on shortest-path method.
Step 2: Recognize Person’s name and make a stop-word list.
Step 3: Based on user-added custom dictionaries, specifically industry-classified dictionaries and stop word dictionaries, stop words are removed from the text information after word segmentation.
Step 4: Quantify the text features after segmentation, and screen out related information such as lending purpose, which is convenient for input to subsequent models for training.

#### 3.2.2 The design of non-text feature module

In this paper, authors search online lending project information table of Renrendai from 2016 to 2018, which is one of the largest P2P lending platforms in China. The credit risk assessment indicators system constructed for borrowers in P2P lending includes 29 evaluation indicators, of which 14 are numerical indicators and 15 are categorical indicators. The indicators are shown in **Table 1**.

**Table 1:** P2P lending borrower credit risk assessment indicators.

First Grade Indicators	Second Grade Indicators
**Personal Information**	A_1_ gender
	A_2_ age
	A_3_ education
	A_4_ marital status
	A_5_ city
**Occupational Information**	A_6_ working field
	A_7_ company scale
	A_8_ income range
	A_9_ working years
**Loan Information**	A_10_ loan amount
	A_11_ annul interest rate
	A_12_ loan term
	A_13_ lending purpose
	A_14_ prepayment rate
	A_15_ guaranty mode
	A_16_ repayment mode
**Historical Loan Information**	A_17_ application number
	A_18_ repayment number
	A_19_ overdue number
	A_20_ successful loan number
	A_21_ total loan
	A_22_ credit limit
	A_23_ overdue amount
	A_24_ unpaid loan principal and interests
	A_25_ serious overdue number
**Other Information**	A_26_ house property (with or without)
	A_27_ house loan (with or without)
	A_28_ vehicle information (with or without)
	A_29_ car loan (with or without)

#### 3.2.3 The design of BP learning algorithm module

Firstly, the model training for the credit risk assessment of borrowers in P2P lending is conducted by transforming textual features into numerical sequences and combining them with non-text feature coding vectors, which are input into the corresponding group of hidden layer neurons. By selecting proper hidden layer transfer function, the sequences are studied through the BP neural network. Secondly, a suitable output layer transfer function is selected, through which the loss function is trained. The weights of neuron are adjusted along the neural network backwards layer by layer, and finally the binary classification result for the credit risk of borrowers in P2P lending is obtained.

**Fig 2:**
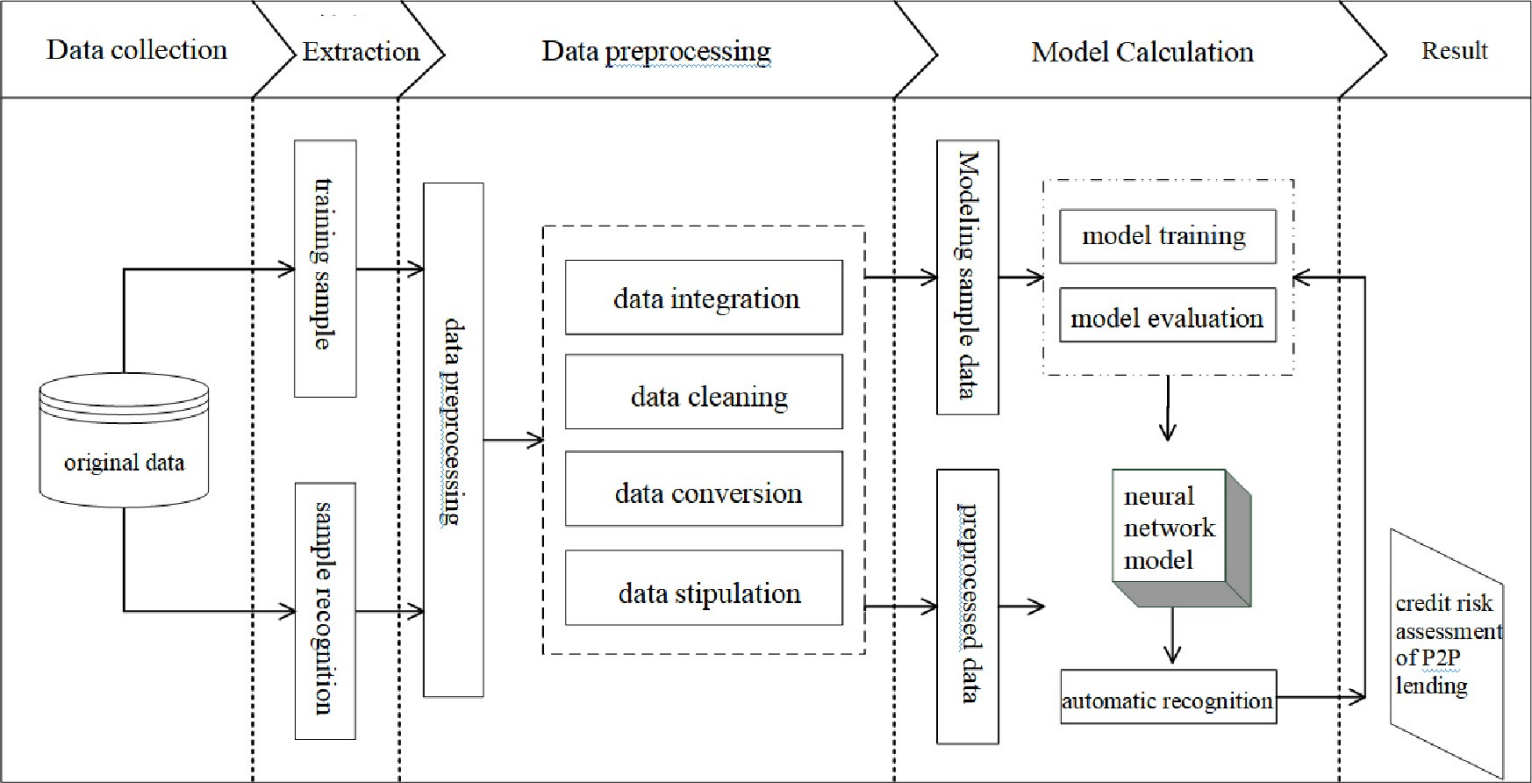
P2P lending borrower credit risk assessment model.

In order to construct a neural network based on BP learning algorithm, authors need to consider following factors during the design process: the layer number of the neural network, the number of neurons contained in each layer (i.e., nodes), transfer function, training method, learning rate, expected error, etc. The learning process of BP neural network mainly consists of four parts: input mode forward propagation, output error backward propagation, cyclic memory training and learning result discriminant. The learning process of BP neural network is shown in **Fig 2**.

## 4. Data source and data preprocessing

### 4.1 Data source

The data used in this paper originate from the information about scattered requests, in which the information and credit of borrowers are evaluated by Renrendai platform, with a total of 10,319 original samples, of which 4,300 are overdue and 6,019 are repaid on time. The original sample collected from Renrendai platform includes 52 indicators, such as project title, request progress, number of participants, number of serious overdue of borrowers, etc. Considering the credit risk assessment indicator system constructed in this paper, two textual indicators and 30 non-textual indicators are selected as the experimental sample. Irrelevant indicators such as bidding progress, number of participants are deleted. In addition, 94 missing values are eliminated to guarantee tidiness and effectiveness of the data.

### 4.2 Data preprocessing

In order to ensure that the input is complete and valid, data preprocessing is required before inputting the data into the model, which mainly includes three steps: categorical data transformation, data normalization and sample set construction. Firstly, authors quantify 15 categorical indicators such as gender, education, marital status, city, and loan description of the borrower and encode each detailed value according to its influence on personal credit assessment in actual work differently. The process of quantification is shown in the Appendix. In addition, since the selected assessment indicators have different dimensions and units, normalization processing is applied to the data. After the normalization process, the data of each indicator is of the same order, and therefore can be comparable, which is suitable for the subsequent comprehensive and comparative analysis of the data. At present, the commonly used normalization processing methods include Z-score normalization, min-max normalization, and function transformation method, etc. In this paper, the credit risk assessment indicators system constructed for borrowers in P2P lending includes 29 evaluation indicators, of which 14 are numerical indicators and 15 are categorical indicators. Different methods are applied to the different attributes of the indicators respectively: Z-score normalization is used to process 14 numerical indicators. One-hot encoding processing is performed on 15 categorical indicators, and the integrated vector obtained from the processing is represented as Smon-1, the representation vector of categorical indicators. In this study, the training data, validation data and test data are divided according to the ratio of 70%, 15% and 15%, which are randomly selected by the neural network model.

## 5. Model design

### 5.1 Network structure parameter settings

After the neural network is determined, authors should first set the network structure parameters, which mainly includes the number of network layers and the number of nodes in each layer. The number of layers in the neural network is determined to be 3, since three-layer BP neural network can realize a mapping from multidimensional unit cube R^m^ to R^n^, which can approximate any rational function. The prediction accuracy of designed network can be adjusted by varying the number of neurons in the hidden layer. The number of input layer nodes depends on the dimensions of the input vector. Based on the credit risk assessment indicators system constructed in this paper, the number of input layer nodes is designed as 29, corresponding to 29 indicators. The number of nodes in the output layer is determined by the abstract model got from the practical problems. The model constructed in this paper only predicts the borrowing behavior corresponding to borrower’s lending project, and the prediction results only conclude two categories- whether the borrower has default risk or not. Therefore, two output nodes are chosen for the output layer. The number of nodes in the hidden layer has a significant impact on the performance of the neural network. Since the design of hidden layer nodes in a neural network is related to its prediction capability, the decision on the number of nodes in the hidden layer is complex and significant. The optimal number of hidden layer neuron nodes should be decided based on repeated trials and test results.

Theoretically, the optimal number of nodes in the hidden layer can be calculated by formula (1) to formula (5):

$$\sum_{i=1}^{n}C_{M}^{i}>k$$ (1)

If

$$i>n_{i}$$ (2)

it is stipulated that,

$$C_{M}^{i}=0$$ (3)

$$M<\sqrt{n+m}+c$$ (4)

$$M=\log_{2}n$$ (5)

Where k represents the amount of samples; M represents the number of hidden layer neurons; n represents the number of input layer neurons; m represents the number of output layer neurons; c represents an arbitrary constant in the interval [28,29].

According to the above formula, set the initial number of nodes in the hidden layer as 7, and then compare learning efficiency and the number of misjudgments corresponding to different hidden layer nodes by trial-and-error testing. Therefore, authors can select the number of nodes in the hidden layer corresponding to the optimal learning efficiency and the number of misjudgments and take it as the number of hidden layer nodes in this model.

### 5.2 Training parameter settings

In this step, authors mainly focus on learning function, training function, activation function, and performance function in the neural network model, as well as parameters such as the training times, error precision, and learning rate. In BP neural network, it is required that the transfer function should be differentiable. Therefore, the Sigmoid function or linear function is generally used as transfer function in BP neural network. A linear function is relatively simple, in which the input is equal to the output. Sigmoid function can be divided into Log-Sigmoid function and Tan-Sigmoid function according to whether its output value contains negative value.

The characteristic of the Log-Sigmoid function is to map the data in the real number range to the interval (0,1). Formula (6) shows the calculation formula for the Log-Sigmoid function.


$$f(x)=\log\;sig(n)=\frac{1}{1+e^{-n}}$$ (6)

Where: *x*∈(−∞, +∞), *f*(*x*)∈(0, 1).

Tan-Sigmoid is a hyperbolic tangent Sigmoid function characterized by mapping data in the real number range to the interval (-1,1). Formula (7) shows the calculation formula for the Tan-Sigmoid function.

$$f(x)=\tan\;sig(n)=\frac{2}{1+e^{-2n}}-1$$ (7)

Where: *x*∈(−∞, +∞), *f*(*x*)∈(−1, 1).

**Fig 3:**
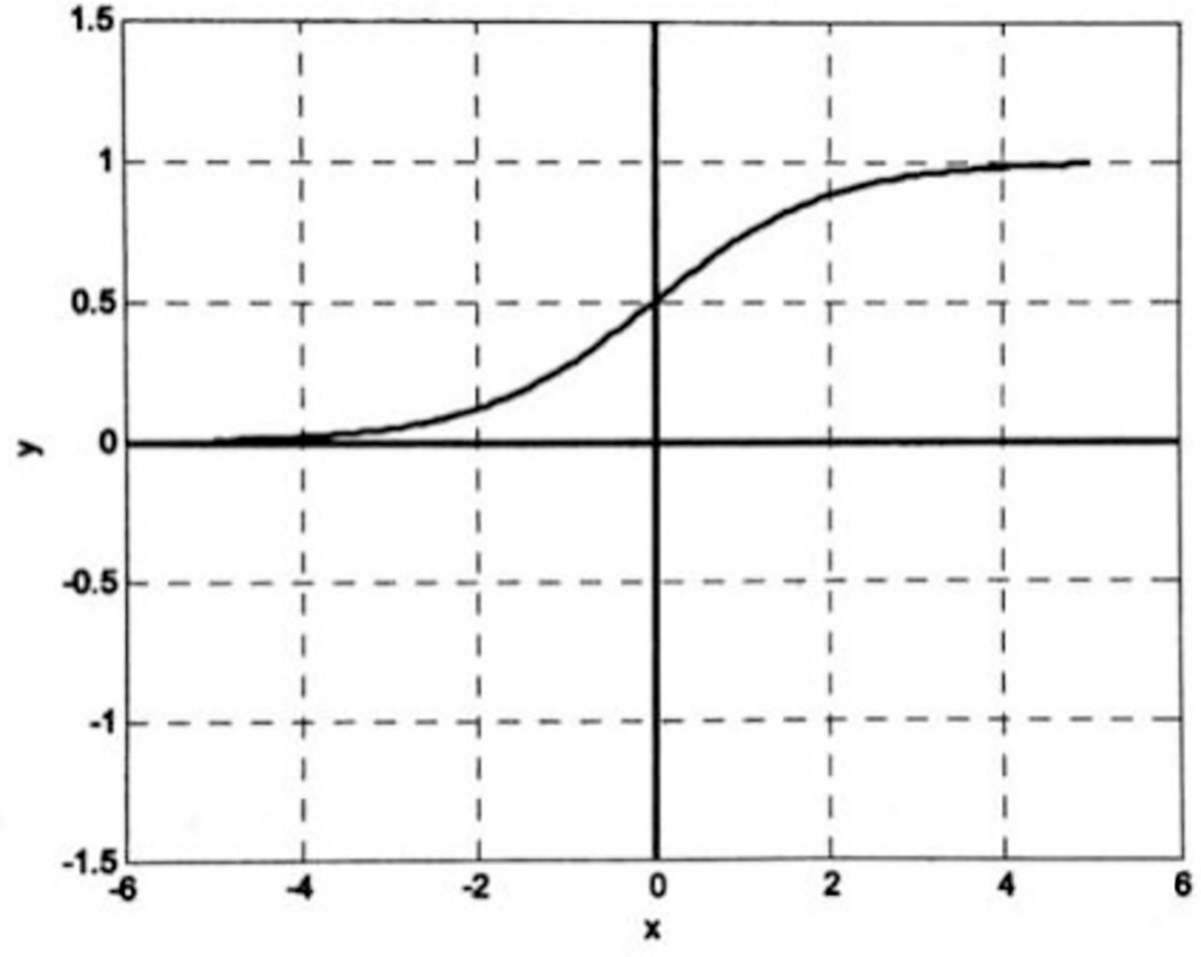
Log-Sigmoid function.

**Fig 4:**
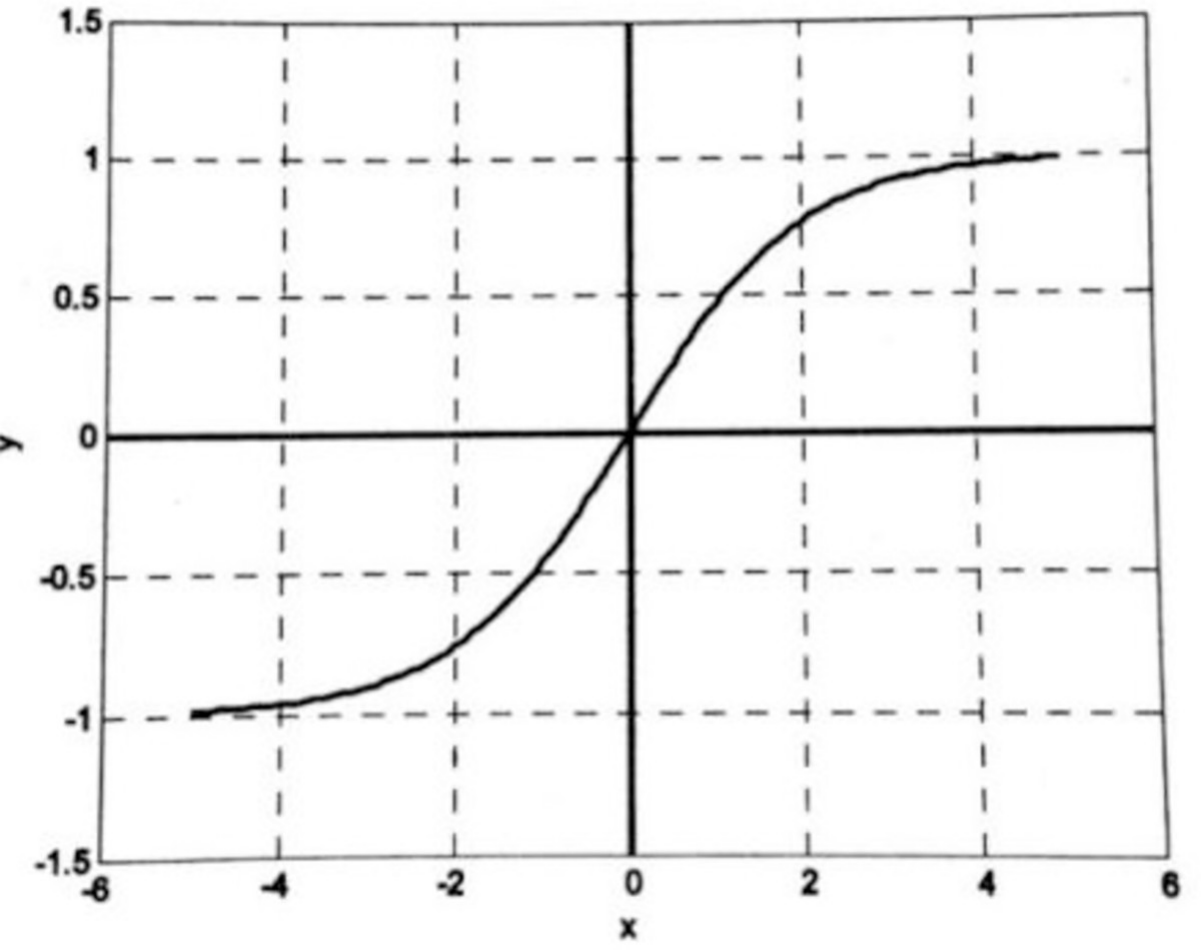
Tan-Sigmoid function.

The curves of the Log-Sigmoid and Tan-Sigmoid functions are shown in **Figs 3** and **4**, respectively [30].

As is shown in Figs 3 and 4, the Sigmoid functions are smooth and differentiable functions, which are capable of mapping input values from the real number range to the interval (0,1) or the interval (-1,1), with nonlinear amplification. Take positive axis as an example, the input signal is small near the origin where the function curve is convex, and the output value y is greater than the input value x. With the input signal increases, the coefficient of nonlinear amplification gradually decreases, and the output value y starts to be smaller than the input value x. It can be found that if the Sigmoid function is applied to the output layer, the output value will be limited to a small range, in the interval (0,1) or interval (-1,1). Thus, the typical design of a BP neural network is to use the Sigmoid function as the transfer function at the hidden layer and use the linear function as the transfer function at the output layer. Our model will also follow the typical BP neural network transfer function selection method.

Standard BP neural networks are trained by means of the steepest descent method. However, there are some defects in this method, with its results rely on the selected initial value. If the function includes many minimums, the model may stuck into local minimum point and fail to reach the global minimum point. To make up the defects of the steepest descent method, the neural network will be trained by the LM algorithm (Levenberg-Marquardt), Scaled Conjugate Gradient, and Bayesian Regularization respectively, and the optimal results will be selected as the training method for the credit risk assessment model of borrowers in P2P lending constructed in this paper.

The error function of neural network trained by the BP algorithm is typically a hypersurface with multiple local minimal points. The initial weight of the neural network determines the starting point of the training on error curved surface, which will directly decide the final convergence point of BP algorithm. The activation function of neurons in BP neural network is generally a origin-symmetric function. So in order to accelerate training process and prevent network paralysis, the initial weights of the neural network should be a evenly distributed small-amount empirical value, which usually in the interval (-1,1) or interval (-2.4/n, +2.4/n), or a random number in even smaller range, where n is the number of neurons in the input layer of the neural network.

The learning rate determines the variable quantity of weights produced during the neural network’s cyclic memory training. In general, the higher the learning rate is, the faster the convergence rate will be, and the more likely it is to oscillate. On the contrary, the lower the learning rate is, the slower the convergence rate will be, and the more stable the system is. To ensure the stability of the system, a lower learning rate is generally preferred, with the range of values located within the interval (0.01, 0.8). In the training of the model, a varying adaptive learning rate can be applied, which can effectively reduce the number of training sessions and training time so as to find the proper learning rate. Therefore, this model will adopt a varying adaptive learning rate.

## 6. Model calculation

In this paper, the LM algorithm (Levenberg-Marquardt), Scaled Conjugate Gradient, and Bayesian Regularization are respectively applied to train the BP neural network. To guarantee logical coherence, the core code of the article is presented in the Appendix.

### 6.1 Training based on the LM algorithm

Authors conduct the training of BP neural network by MATLAB programming. Firstly, create a BP neural network by feedforwardnet function in MATLAB neural network toolbox. Then, train the BP neural network using net.trainFcn = ’trainlm’ based on LM algorithm. By trial-and-error testing, the optimal hidden layer nodes of the neural network model is determined in the process of training the neural network by LM algorithm.

**Fig 5:**
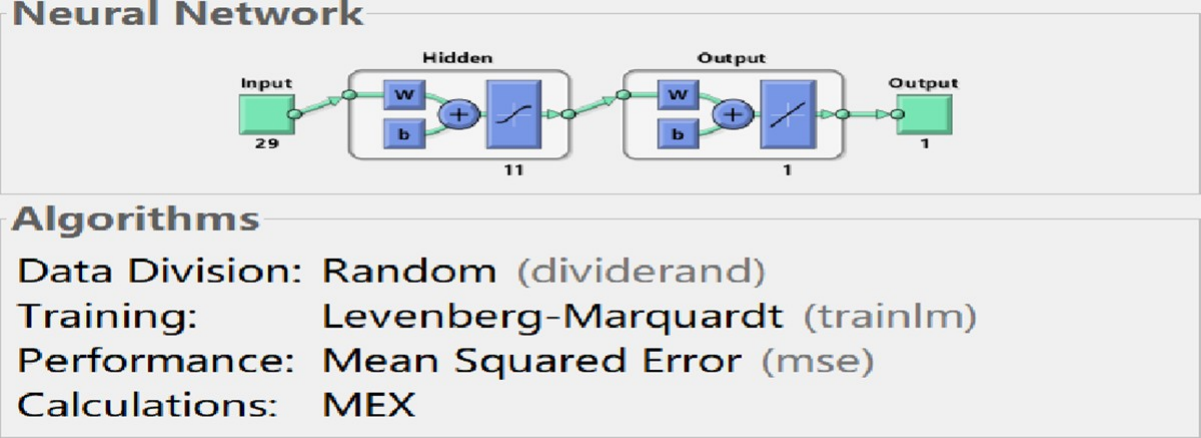
BP neural network structure (LM).

By comparing the accurate rate of each hidden node, authors can see that when the number of hidden layer nodes is set to 12, the model has the maximum mean square error, which is 0.000632. When the number of hidden nodes is set to 11, the model has the minimum mean square error, which is 8.71e-15 (see the **Table 2**). Therefore, when the neural network is trained by means of LM algorithm, the input layer node of the optimal model is 29, the hidden layer node is 11, and the output layer node is 1. The structure diagram of the neural network model is shown in **Fig 5**.

**Table 2:** Comparison of prediction with different hidden Layer Nodes (LM).

Hidden layer nodes	Epoch	Performance	Gradient	Validation Checks
**7**	120	1.78e-13	3.81e-09	4
**8**	314	4.49e-09	2.02e-05	6
**9**	207	1.50e-12	9.98e-08	0
**10**	565	8.75e-12	9.96e-08	0
**11**	27	8.71e-15	8.92e-08	0
**12**	25	0.000632	0.0655	6
**13**	24	0.000606	0.00223	6

**Fig 6:**
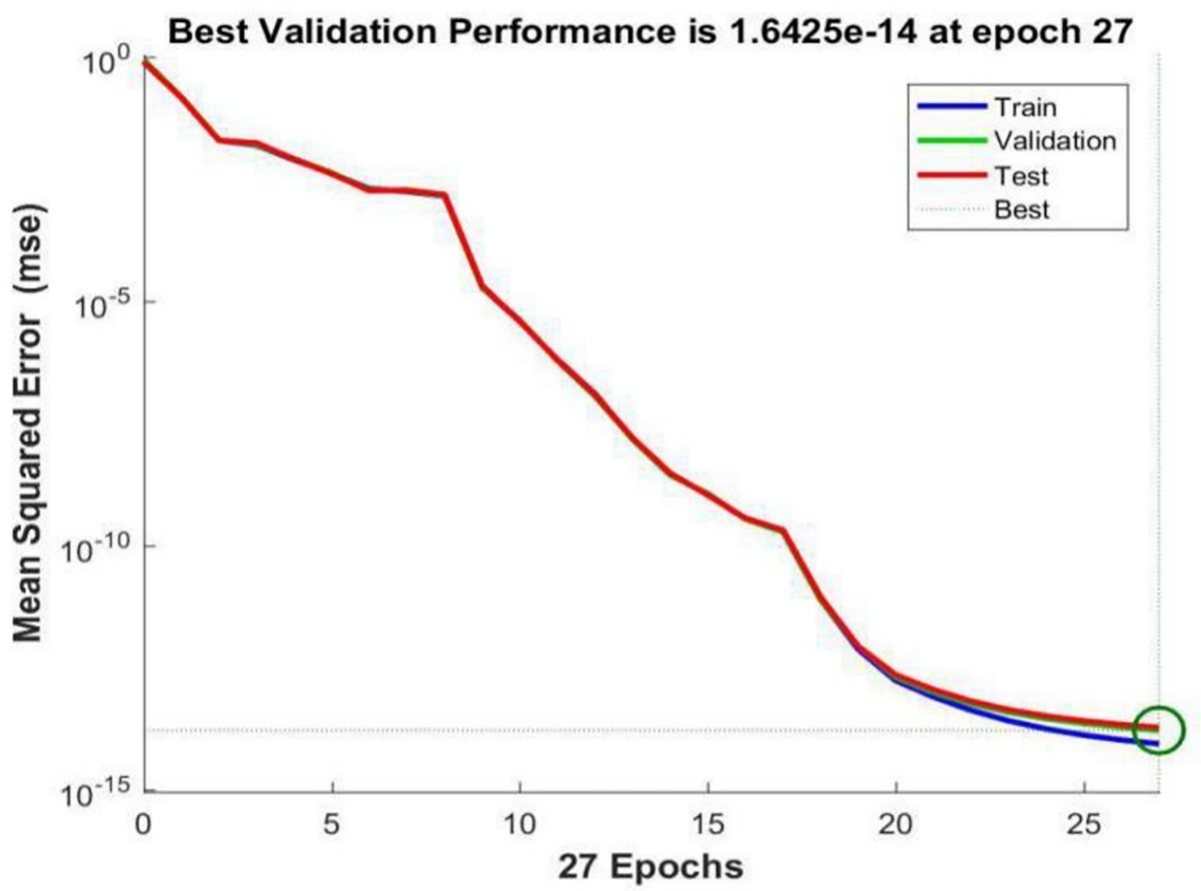
Trainlm performance graph.

**Fig 6** shows the performance curve of BP neural network based on LM algorithm where the input layer node is 29, the hidden layer node is 8, and the output layer node is 1. From the figure, it can be seen that when the iteration number reaches 27, the neural network perform best in testing error, which is 1.6425e-14.

### 6.2 Training based on scaled conjugate gradient

Authors conduct the training of BP neural network by MATLAB programming. The BP neural network is trained by net.trainFcn = ’trainlm’ based on Scaled Conjugate Gradient in MATLAB neural network toolbox. By trial-and-error testing, authors can firstly determine the optimal hidden layer nodes of the neural network model in the process of training based on Scaled Conjugate Gradient.

**Fig 7:**
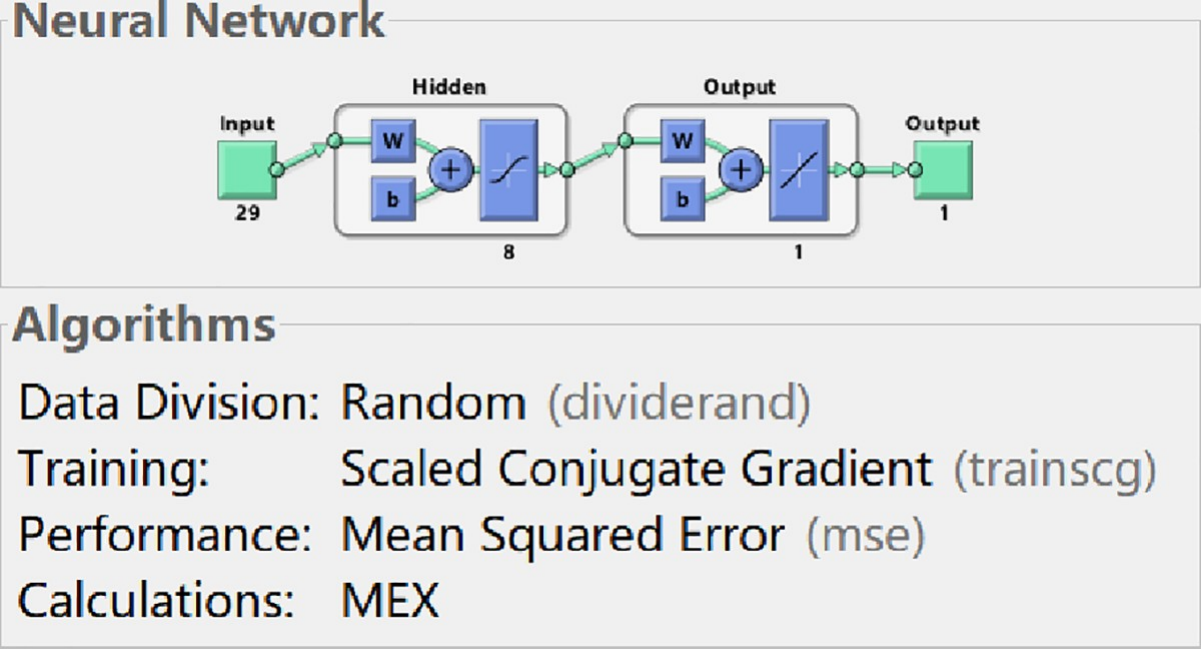
BP neural network structure (SCG).

By comparing the accurate rate of each hidden node, authors train the neural network based on Scaled Conjugate Gradient, the results are as follows: when the number of hidden layer nodes is set to 12, the model has the maximum mean square error, which is 0.00167. When the number of hidden nodes is set to 8, the model has the minimum mean square error, which is 0.000707 (see the **Table 3**). Therefore, when the neural network is trained by means of Scaled Conjugate Gradient, the input layer node of the optimal model is 29, the hidden layer node is 8, and the output layer node is 1. The structure diagram of the neural network model is shown in **Fig 7**.

**Table 3:** Comparison of prediction with different hidden layer nodes (SCG).

Hidden layer nodes	Epoch	Performance	Gradient	Validation Checks
**7**	172	0.000734	0.00148	6
**8**	190	0.000707	0.000655	6
**9**	142	0.00122	0.00349	6
**10**	179	0.000906	0.00158	6
**11**	229	0.000783	0.00156	6
**12**	96	0.00167	0.00519	6
**13**	142	0.000960	0.00176	6
**14**	174	0.000851	0.00399	6

**Fig 8:**
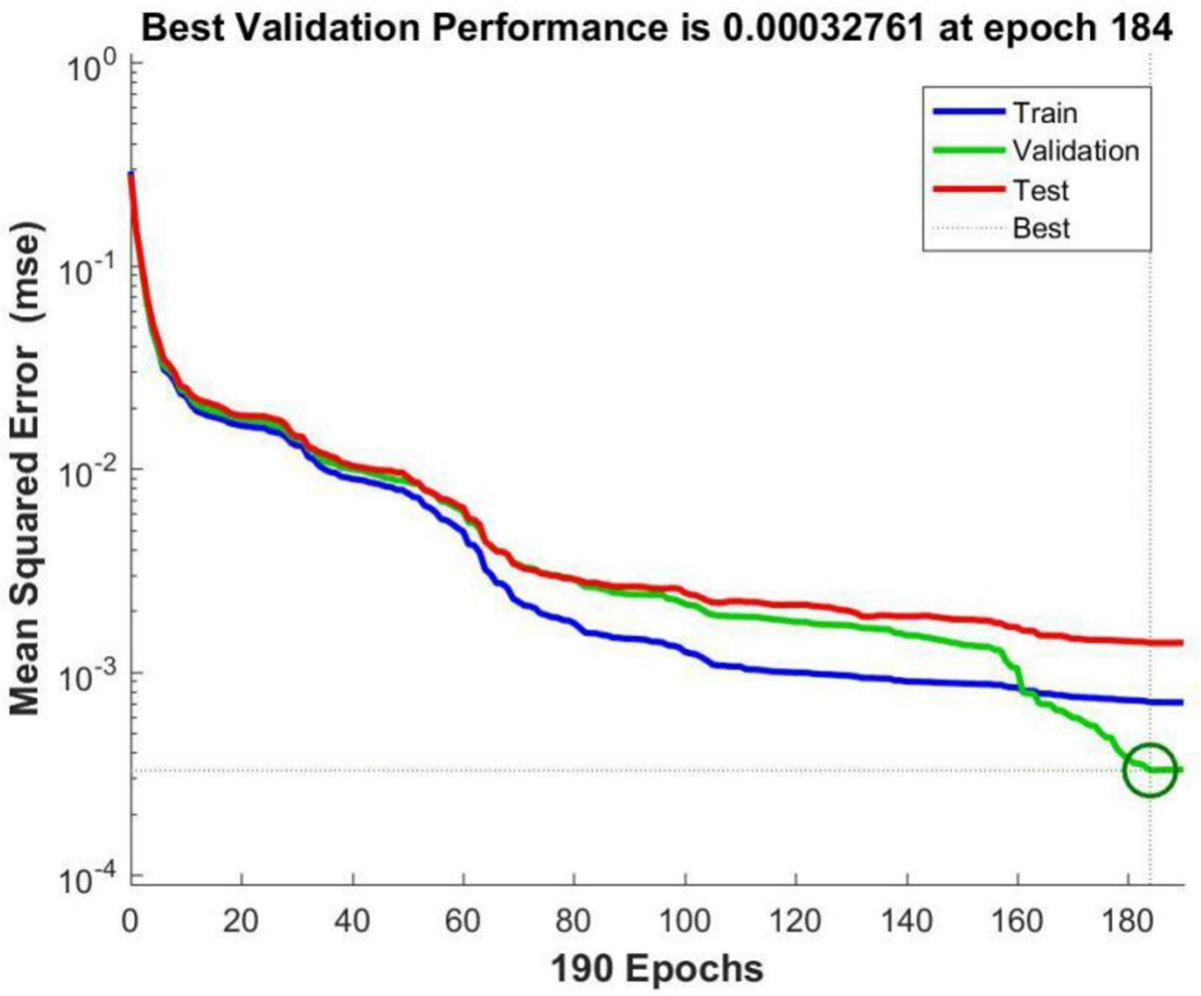
Trainscg performance graph.

**Fig 8** shows the performance curve of BP neural network based on Scaled Conjugate Gradient where the input layer node is 29, the hidden layer node is 8, and the output layer node is 1. From the figure, it can be seen that when the iteration number reaches 184, the neural network perform best in testing error, which is 0.00032761.

### 6.3 Training based on Bayesian Regularization

Authors conduct the training of BP neural network by MATLAB programming. The BP neural network is trained by net.trainFcn = ’trainlm’ based on Bayesian Regularization in MATLAB neural network toolbox. By trial-and-error testing, Authors can firstly determine the optimal hidden layer nodes of the neural network model in the process of training based on Bayesian Regularization.

**Fig 9:**
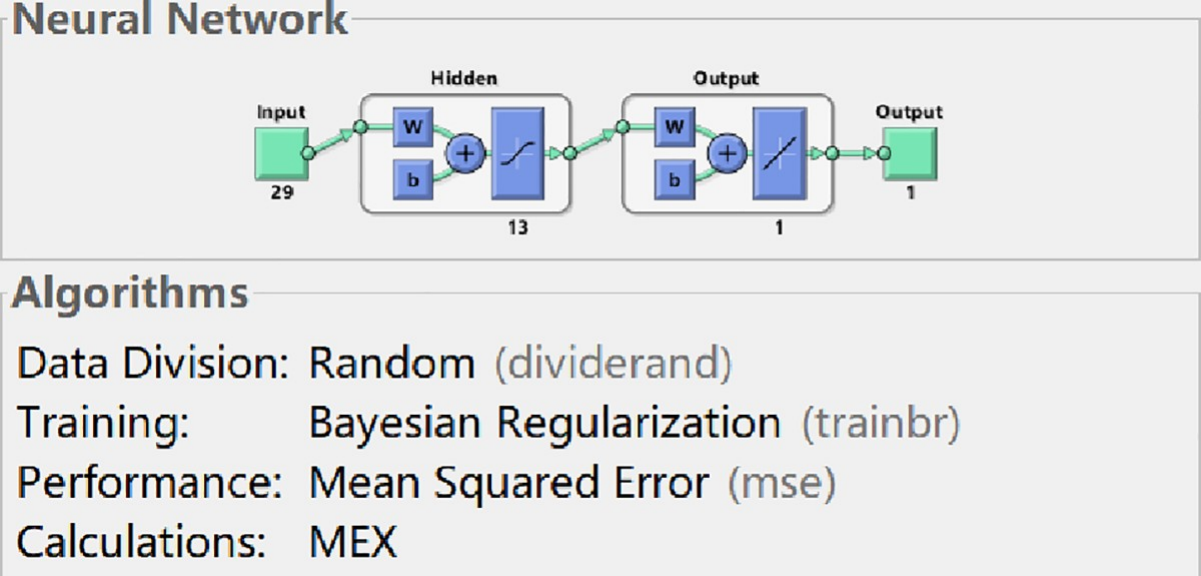
BP neural network structure (BR).

By comparing the accurate rate of each hidden node, Authors train the neural network based on Bayesian Regularization, the results are as follows: when the number of hidden layer nodes is set to 11, the model has the maximum mean square error, which is 1.85e-10. When the number of hidden nodes is set to 13, the model has the minimum mean square error, which is 1.96e-13 (see the **Table 4**). Therefore, when the neural network is trained by means of Bayesian Regularization, the input layer node of the optimal model is 29, the hidden layer node is 13, and the output layer node is 1. The structure diagram of the neural network model is shown in **Fig 9**.

**Table 4:** Comparison of prediction with different hidden layer nodes (BR).

Hidden layer nodes	Epoch	Performance	Gradient	Validation Checks
**7**	190	5.98e-12	3.70e-07	0
**8**	92	8.43e-12	8.83e-06	0
**9**	378	4.50e-12	2.03e-05	0
**10**	462	4.53e-12	2.34e-06	0
**11**	178	1.85e-10	8.79e-07	0
**12**	373	2.42e-12	1.57e-05	0
**13**	308	1.96e-13	1.13e-08	0
**14**	141	1.92e-12	2.03e-08	0

**Fig 10:**
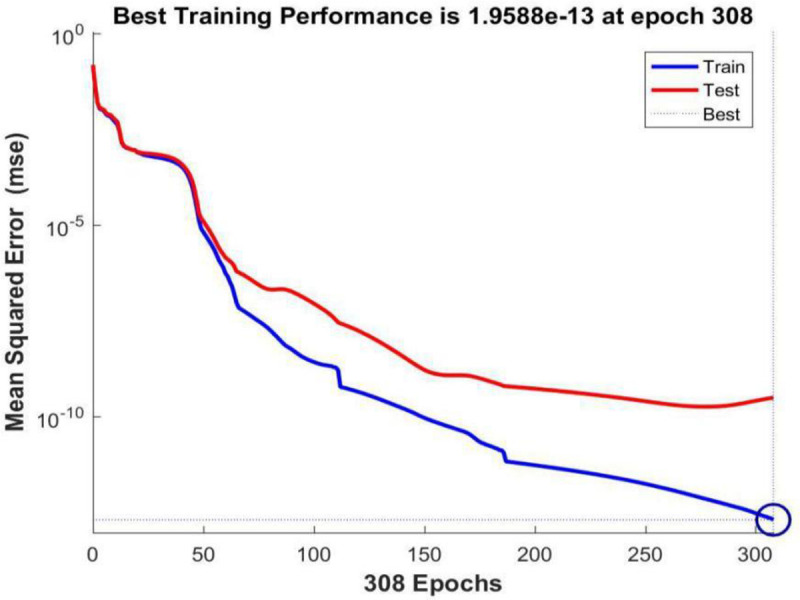
Trainbr performance graph.

**Fig 10** shows the performance curve of BP neural network based on Bayesian Regularization where the input layer node is 29, the hidden layer node is 13, and the output layer node is 1. From the figure, it can be seen that when the iteration number reaches 308, the neural network perform best in testing error, which is 1.9588e-13.

Comparing the performance curves of neural networks based on LM algorithm (Levenberg-Marquardt), Scaled Conjugate Gradient, and Bayesian Regularization as training functions, the net work model based on LM algorithm perform best, with only 27 iterations it achieve best testing error result. And the number of iterations and the best testing error are both smaller than those in models based on Scaled Conjugate Gradient and Bayesian Regularization. Therefore, LM algorithm is characterized by fast calculation speed and good prediction ability. So authors choose LM algorithm as the training method for the neural network model, in which the input layer node is 29, the hidden layer node is 11, and the output layer node is 1.

### 6.4 Validation

**Fig 11:**
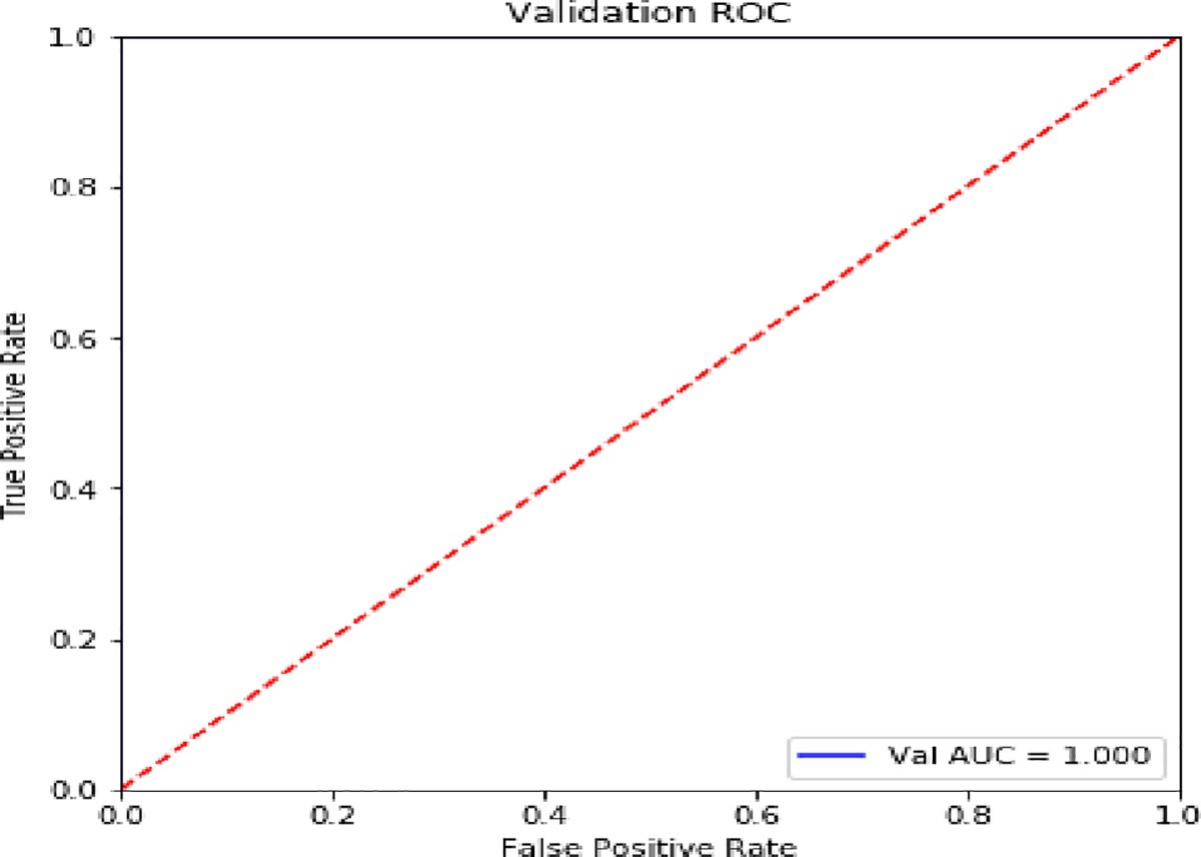
ROC curve.

**Fig 12:**
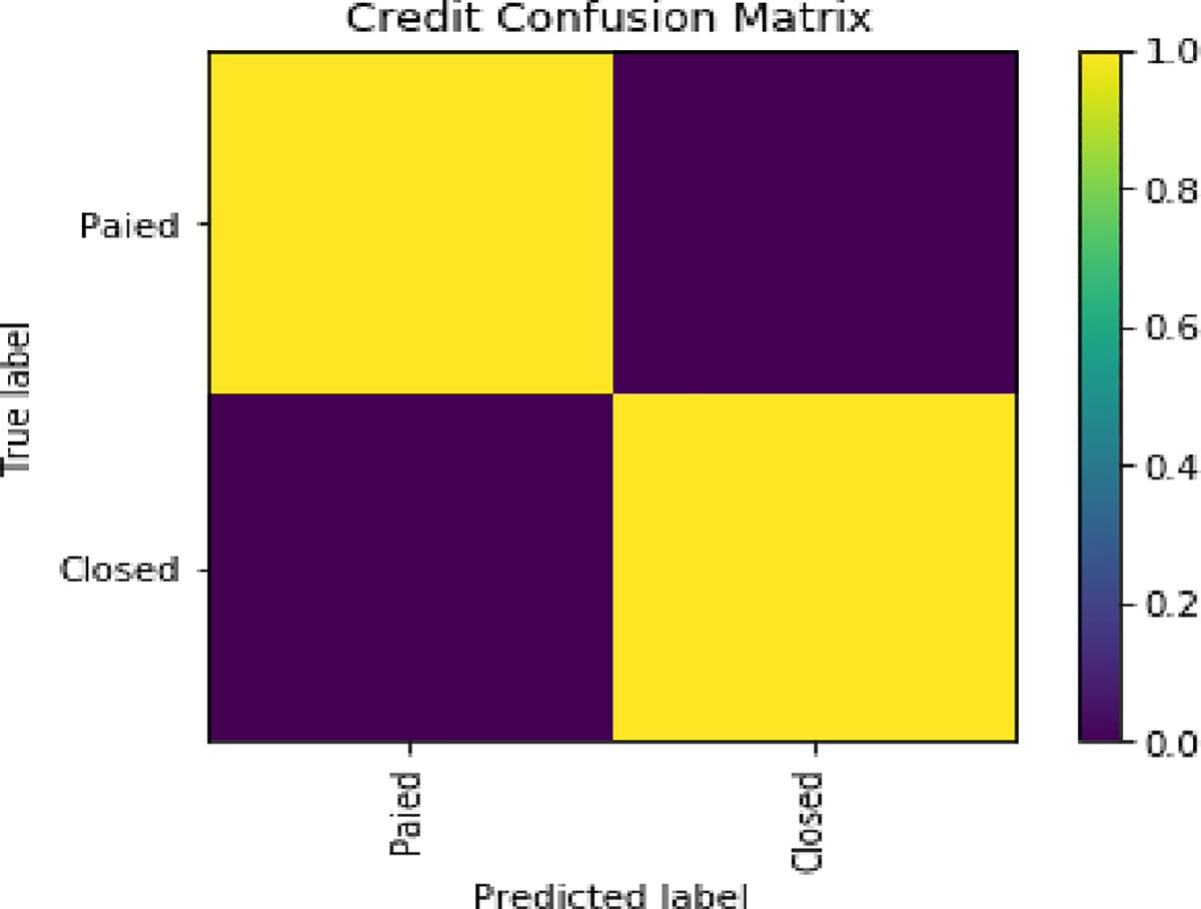
Confusion matrix.

In this paper, precision, recall, F1-score and confusion matrix are used as measurement criteria. The result is shown in **Table 5**, **Figs 11** and **12**.

**Table 5:** Comparison of validation.

	Precision	Recall	F-Score
**Overdue (Default)**	1	1	1
**Paid on time (Implementation)**	1	11	1

## 7. Conclusion

To build a personal credit assessment system of P2P lending that is accurate and reasonable can avoid the occurrence of borrower credit risk to some extent, enhance the monitoring and prediction of borrower credit risk, and therefore reduce borrower default behavior. Based on the current research status on credit risk assessment models for borrowers in P2P lending at home and abroad, this paper combine with existing data from the online lending industry in China, and the basic idea of standard BP neural networks. The algorithm process is deduced and verified in detail, and the main steps are as follows:

(1) On the basis of the 2017 edition of Industrial classification for national economic activities and the gazetteer encyclopedia of national provinces, cities, counties and four-level administrative division the Ansj tokenizer is applied to segment and extract the textual information from the borrower’s loan information, including lending purpose mentioned in the loan title and loan description, which is coded into character information available for input and added to the credit risk indicator system for borrowers in P2P lending.

(2) Based on the credit risk indicator system, authors apply BP algorithm to establish a neural network model and to assess the risk of P2P online lending project information. Therefore, authors construct a BP neural network, in which the input layer node is 9, the hidden layer node is 11, and the output layer node is 1. The neural network is trained by LM algorithm and used as the credit risk assessment model for borrower behavior in P2P lending. Credit risk assessment model. Since authors take borrower’s historical borrowing information (such as the number of successful loans, the number of overdue, etc.) into consideration when selecting indicator system, it can predict the borrowing behavior more accurately based on the borrower’s historical performance.

An effective borrower credit risk management system can not only help control the bad debt rate of online lending platforms and reduce operation costs, but also enhance investors’ confidence and improve the public image of the platform. Therefore, the research in this paper has practical guiding significance for P2P lending platforms. On the one hand, for platforms that have already established a borrower credit risk management system, the management system of platform can be updated according to the credit risk assessment indicator system for borrowers in P2P lending constructed in this paper. Text information such as loan descriptions and loan titles can be integrated into the platform’s self-built assessment system by drawing on the processing method of text information in this paper, so as to have a more comprehensive and accurate review of borrower credit risk. On the other hand, for online lending platforms that have not yet established the borrower credit risk management system, the platform can build an effective borrower credit risk management system based on the results of this paper’s research combining with their own characteristics and thus effectively manage the credit risks of the platform’s borrowers.

## Appendix A

Data description (see Table 6)

**Table 6:** P2P lending borrower credit risk assessment indicators.

First Grade Indicators	Second Grade Indicators
**Personal Information**	A_1 =_ gender
	A_2 =_ age
	A_3 =_ education
	A_4 =_ marital status
	A_5 =_ city
**Occupational Information**	A_6 =_ working field
	A_7 =_ company scale
	A_8 =_ income range
	A_9 =_ working years
**Loan Information**	A_10 =_ loan amount
	A_11 =_ annul interest rate
	A_12 =_ loan term
	A_13 =_ lending purpose
	A_14 =_ prepayment rate
	A_15 =_ guaranty mode
	A_16 =_ repayment mode
**Historical Loan Information**	A_17 =_ application number
	A_18 =_ repayment number
	A_19 =_ overdue number
	A_20 =_ successful loan number
	A_21 =_ total loan
	A_22 =_ credit limit
	A_23 =_ overdue amount
	A_24 =_ unpaid loan principal and interests
	A_25 =_ serious overdue number
**Other Information**	A_26 =_ house property (with or without)
	A_27 =_ house loan (with or without)
	A_28 =_ vehicle information (with or without)
	A_29 =_ car loan (with or without)

**Fig 13:**
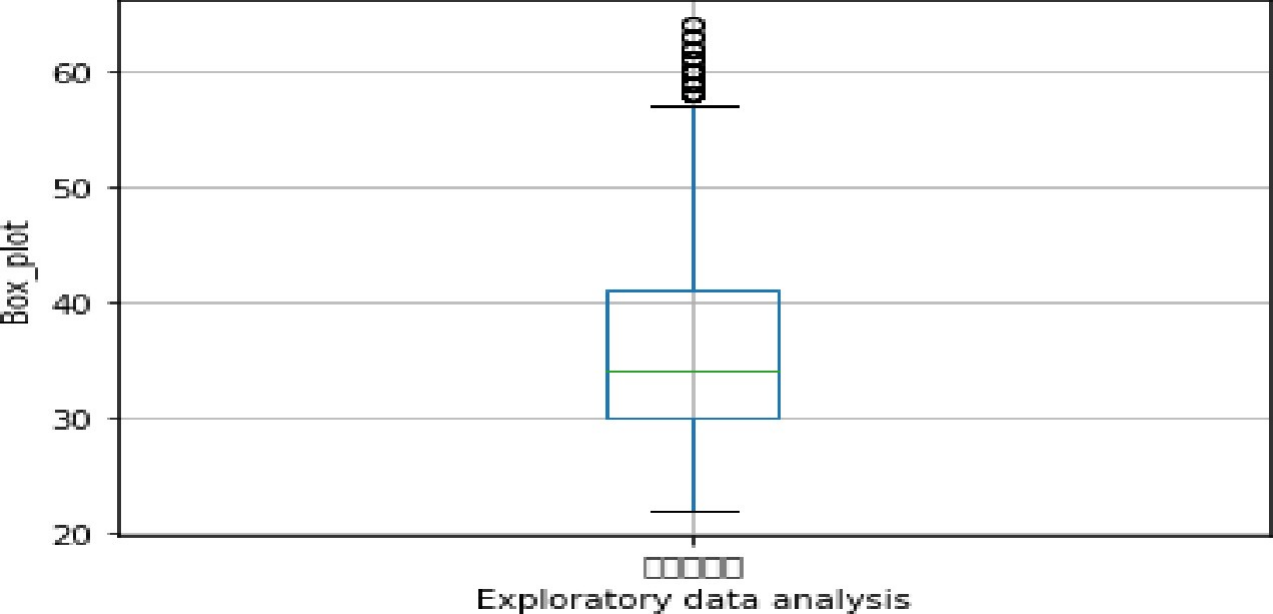
Exploratory data analysis for age (A2).

**Fig 14:**
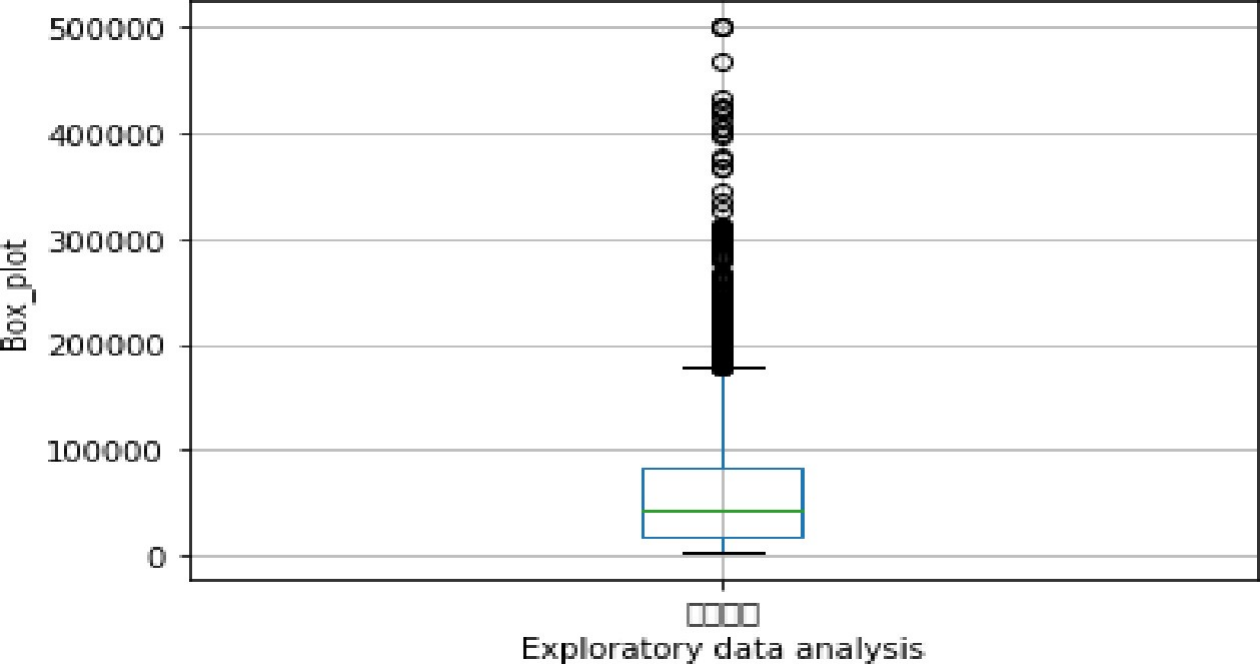
Exploratory data analysis for loan amount (A10).

**Fig 15:**
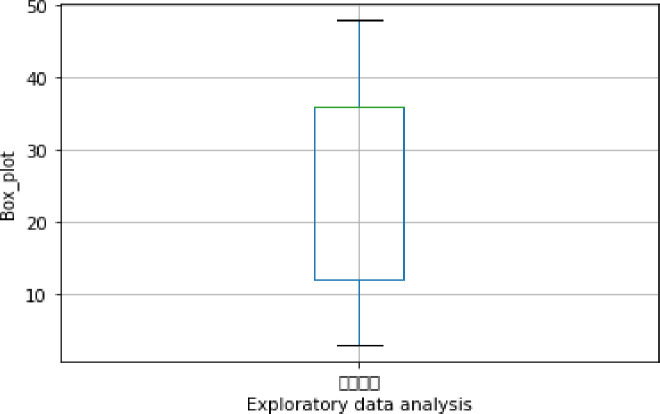
Exploratory data analysis for loan term (A12).

**Fig 16:**
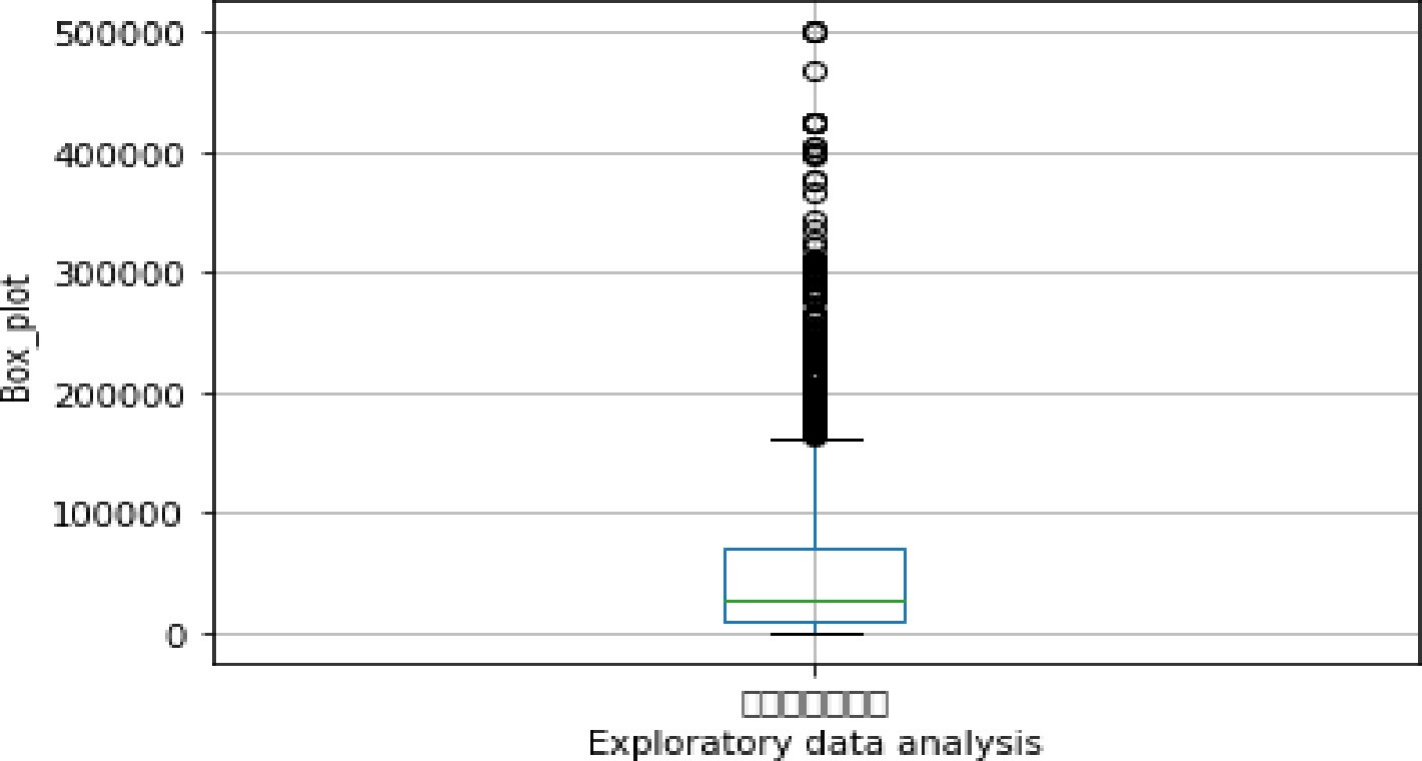
Exploratory data analysis for credit limit (A22).

Exploratory data analysis (see Figs 13–16)

## Appendix B

The authors compared the BP model with competitive models. And the paper used SVM and random forest as model comparison. The result shows in Table 7.

**Table 7:** Compared the BP model with competitive models.

BP	999348109517601%
SVM	999348109517601%
Random Forest	100%

## Appendix C

The paper processed data analysis with five step.

**Fig 17:**
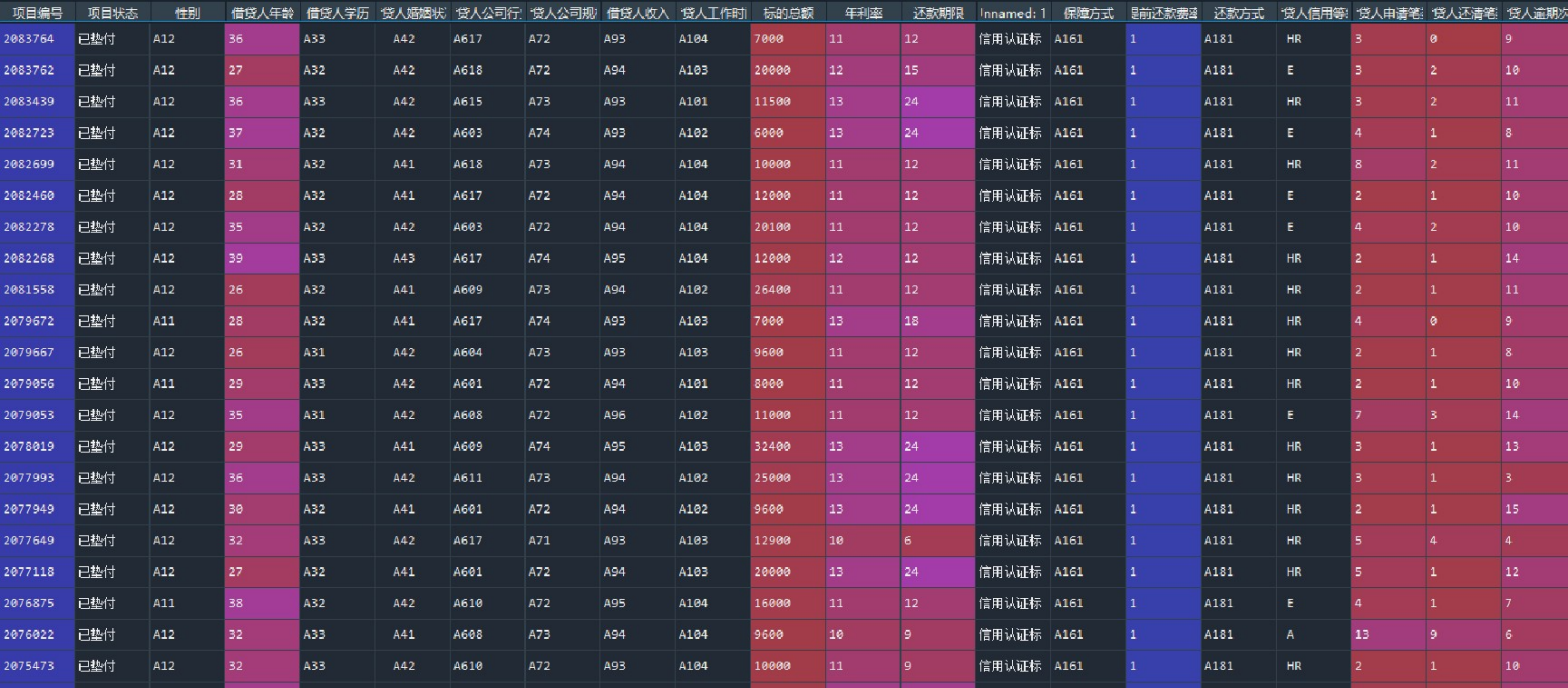
First read Excel data.

Step one: First read Excel data (see Fig 17)

Step two: Label making

The default sample is regarded as label 0, and the paid sample is regarded as label 1.

Step three: Data standardization

**Fig 18:**
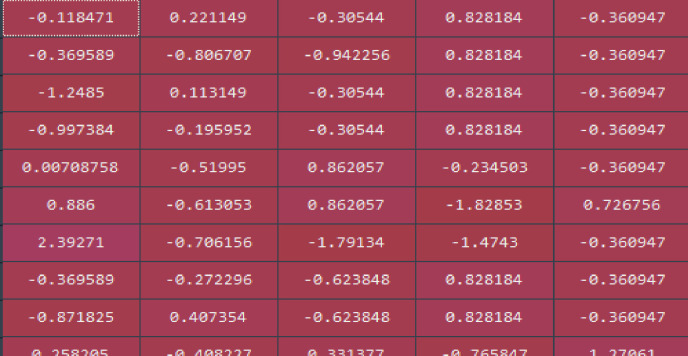
Data standardization.

The discrete data is expressed as 0–1 range, and the large range data is standardized to 0–1 range (see Fig 18).

Step four: Neural network training

Step five: Neural network test results
